# Assessment of Selected Endothelial Damage Biomarkers in the Determination of Endothelial Damage in Cats With Gingivostomatitis

**DOI:** 10.1002/vms3.70525

**Published:** 2025-07-28

**Authors:** Saadet Gözde Korkmaz, Mahmut Ok

**Affiliations:** ^1^ Department of Internal Medicine Faculty of Veterinary Medicine Selcuk University Konya Turkey

**Keywords:** cat, endothelial biomarker, gingivostomatitis, haematological profile

## Abstract

**Background:**

Feline gingivostomatitis is a chronic disease of domestic cats characterised by inflammatory lesions along the gingiva and oral cavity.

**Objectives:**

The aim of this study was to investigate oral vascular endothelial glycocalyx damage in gingivostomatitis caused by viral infections and other causes in cats using selected biomarkers of endothelial damage.

**Methods:**

The material of this study consisted of 55 cats with gingivostomatitis and 15 healthy cats of different age, breed and sex. A total of 34 of 55 cats with gingivostomatitis had viral infections, whereas the cause of 21 cats could not be determined. Viral diseases were diagnosed by clinical findings and rapid antigen tests. Haemogram analysis was performed from blood samples. Serum endothelin‐1 (ET‐1), syndecan‐1 (SDC‐1), myeloperoxidase‐ANCA (MPO‐ANCA) and interleukin‐6 (IL‐6) biomarker concentrations were measured using feline‐specific commercial ELISA kits. Cats were treated medically for gingivostomatitis.

**Results:**

Although 49 cats recovered with the treatment, 6 cats died. There was a significant increase in serum ET‐1 and SDC‐1 concentrations and no difference in serum MPO‐ANCA and IL‐6 concentrations in cats with gingivostomatitis. In addition, total leukocyte (WBC), granulocyte (GRA) and monocyte (MON) counts were increased in cats with gingivostomatitis.

**Conclusion:**

Serum levels of ET‐1 and SDC‐1, which are biomarkers of vascular endothelial damage, were increased in cats with gingivostomatitis. It was shown that vascular endothelial damage occurs in gingivostomatitis and that the biomarkers ET‐1 and SDC‐1 can be used to detect this damage and have a reliable diagnostic value.

## Graphical abstract

In this study, biomarkers of vascular endothelial damage were investigated in cats with gingivostomatitis. It has been shown that vascular endothelial damage occurs in gingivostomatitis and ET‐1 and SDC‐1 biomarkers can be used to detect this damage and have a reliable diagnostic value.

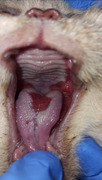

## 1 Introduction

Feline chronic gingivostomatitis (FCGS) is a painful and debilitating oral disease in cats, characterised by severe, chronic, bilateral inflammation of the gums, labial mucosa and caudal oral mucosa (Winer et al. 2016; Druet and Hennet 2017; Ploypetch et al. 2025). Ulcerative or ulceroproliferative lesions are often present. Ulceration of the tongue and palate may also be present. FCGS is also known as caudal stomatitis due to the formation of inflammatory lesions in the caudal oral mucosa. The presence of caudal stomatitis distinguishes FCGS from other feline oral diseases (Hennet et al. 2011; Druet and Hennet 2017; Kim et al. 2023; Soltero‐Rivera et al. 2023). Chronic gingivostomatitis in cats or lymphoplasmacytic is a severe, immune‐mediated, the oral mucosal inflammatory (Kuek et al. 2007; Winer et al. 2016; Ploypetch et al. 2025). Multi factors play a role in the development of chronic gingivostomatitis in cats. These include viruses, breed, environmental factors, bacterial plaque and dental disease. The cause of FCGS is unknown but may be related to an altered immune response to oral antigenic stimulation caused by *feline leukaemia virus* (FeLV), *feline immunodeficiency virus* (FIV), *Feline calicivirus* (FCV), *Feline herpesvirus‐1* (FHV‐1) and *Bartonella* (Quimby et al. 2008; Lommer 2013;Winer et al. 2016; Peralta and Carney 2019). The clinical signs of cats with chronic gingivostomatitis include moderate or severe pain, anorexia, halitosis, dysphagia, weight loss and abnormal behaviour (Druet and Hennet 2017). FeLV causes immunosuppression, myelosuppression, stomatitis, pleuritis, pleural fluid, neoplasia and neurological disease in cats (Dokuseylul et al. 2016; da Costa et al. 2017; Ludwick and Clymer 2019). FIV can cause chronic gingivostomatitis, chronic rhinitis, lymphadenopathy, immune‐mediated glomerulonephritis and weight loss as a result of immunodeficiency or immunostimulation (Dokuseylul et al. 2016; Lee et al. 2020). FCV can cause conjunctivitis, rhinitis, oral ulcers and pneumonia in cats (Hurley and Sykes 2003; Nelson and Couto 2019).

The endothelial glycocalyx (EGC) is a dynamic layer that regulates coagulation, leukocyte surface adhesion and mechanotransduction by interacting with the endothelium and plasma components surrounding the luminal surface of the vasculature (Yang et al. 2018). Various diseases, such as sepsis (Uchimido et al. 2019; Naseri et al. 2020; Yamako‐Tojo 2020), kidney injury (Padberg et al. 2014; Libório et al. 2015), cardiovascular disease (Kim et al. 2017) and lung disease (Okada et al. 2021; Suzuki et al. 2021), have been reported to cause EGC damage (Suzuki et al. 2022; Franceković and Gliemann 2023).

An increase in serum endothelin‐1 (ET‐1) concentration was observed in premature calves with respiratory distress compared to premature calves without respiratory distress and healthy calves, and the ET‐1 marker was found to be prognostic for mortality in premature calves (Ider et al. 2021). İsmailoğlu (2023) found a significant increase in serum ET‐1 level in acute respiratory distress syndrome in cats and associated this increase with damage to the epithelium and endothelium of the lung. It has been reported that serum concentrations of syndecan‐1 (SDC‐1), ET‐1 and vascular endothelial growth factor‐A (VEGF‐A) are increased in cats with haemotropic mycoplasmosis. Furthermore, it has been suggested that SDC‐1 and ET‐1 can be considered diagnostic biomarkers, whereas VEGF‐A can be considered a prognostic biomarker (Ider et al. 2024). SDC‐1 is a heparan sulphate/chondroitin sulphate proteoglycan (PG) found on the surface of cells and in the extracellular matrix (Cao et al. 2019). SDC‐1 concentration has been shown to be the main indicator of EGC damage in humans and rats (Torres‐Filho et al. 2016; Barber et al. 2021).

Proteinase 3‐ANCA (PR3‐ANCA) and myeloperoxidase‐ANCA (MPO‐ANCA) are biomarkers used to detect vasculitis or granulomatous vasculitis (Schönermarck et al. 2015). Gulersoy et al. (2023) found no difference in serum MPO‐ANCA and PR3‐ANCA concentrations in cats with feline infectious peritonitis (FIP) compared to healthy cats. Interleukin‐6 (IL‐6) is a cytokine with multiple biological activities. During infection and injury, the biosynthesis of IL‐6 is stimulated in an emergency and the response plays a role in activating host defence and immunity (Wiegertjes et al. 2019). It has been suggested that IL‐6 may also be used to determine organ dysfunction, morbidity and mortality in patients with acute respiratory distress syndrome (Darif et al. 2021).

This study was designed with the hypothesis that vascular endothelial damage caused by immune‐mediated oral mucosal inflammation in gingivostomatitis caused by feline FeLV, FCV, FIV and FHV‐1 infections and other causes can be detected by ET‐1, SDC‐1, MPO‐ANCA and IL‐6 biomarkers. The aim of this study was to investigate oral vascular endothelial damage in gingivostomatitis caused by feline viral infections and other causes using selected biomarkers of endothelial damage.

## 2 Materials and Methods

### 2.1 Animals

This study was carried out on 55 cats with gingivostomatitis (aged 1–10 years), breeds and sexes (29 males, 26 females) and 15 healthy cats (aged 1–7 years), breeds and sexes (7 males, 8 females) (Table 1). The authors confirm that the ethical policies of the journal, as noted on the journal's guidelines page, have been adhered to and the appropriate ethical review committee approval has been received. The study was conducted with the approval of the Selcuk University Veterinary Faculty Experimental Animal Production and Research Center Ethics Committee (SUVDAFEK) with decision number 2024/008.

**TABLE 1:** Breed, sex, age, survival and antigen test positivity of cats with viral gingivostomatitis.

Case no	Breeds	Sex	Age	Survival	FİV	FeLV	FCV	FHV‐1
1	Tabby	Male	5	Survival	−	**+**	−	−
2	British	Female	2	Survival	−	**+**	−	−
3	Tabby	Female	4	Survival	+	−	−	−
4	Tabby	Female	1	Survival	−	**+**	−	−
5	Tabby	Male	2	Survival	−	+	−	−
6	Scottish fold	Male	6	Survival	+	−	−	−
7	Tabby	Male	2	Survival	+	−	−	−
8	Tabby	Female	6	Survival	−	−	+	−
9	Tabby	Male	6	Survival	−	+	−	−
10	Tabby	Male	7	Survival	−	+	−	−
11	British shorthair	Male	2	Survival	−	+	−	−
12	British shorthair	Female	1	Nonsurvival	−	+	−	−
13	Tabby	Male	1	Survival	−	−	+	−
14	Tabby	Male	1	Survival	−	+	−	−
15	British	Male	1	Survival	−	−	+	−
16	Scottish fold	Female	5	Nonsurvival	+	−	−	−
17	Tabby	Female	2	Survival	−	+	−	−
18	Tabby	Female	6	Survival	+	−	−	−
19	Tabby	Male	5	Survival	−	−	+	−
20	Tabby	Female	1	Survival	−	+	−	−
21	Tabby	Male	10	Nonsurvival	+	−	−	−
22	Tabby	Female	4	Nonsurvival	+			
23	British shorthair	Female	1	Survival		+	−	−
24	Tabby	Female	8	Nonsurvival	+	−	−	−
25	Tabby	Male	9	Survival		+		
26	Tabby	Male	6	Survival	−	+	−	−
27	Tabby	Male	1	Nonsurvival	−	+	−	−
28	Tabby	Male	1	Survival	−	−	+	−
29	Tabby	Male	1	Survival	−	−	+	−
30	Tabby	Male	4	Survival	+	−	−	−
31	Tabby	Female	10	Survival	+	−	−	−
32	Tabby	Female	4	Survival	−	+	−	−
33	Tabby	Female	2	Survival	−	−	−	+
34	Tabby	Male	3	Survival	−	+	−	−

Abbreviations: FCV, Feline calicivirus; FeLV, feline leukaemia virus; FHV‐1, feline herpesvirus‐1; FIV, feline immunodeficiency virus.

### 2.2 Selection of Cats With Gingivostomatitis

The anamnesis, sexes, breeds, age and the number of days of the problem were recorded for the cats brought with the complaint of gingivostomatitis. The anamnesis revealed that most of the cats in the study had complained of gingivostomatitis for a month, with some experiencing several recurrences after treatment. Gingival, oral cavity and systemic examinations were performed. For cats with gingivostomatitis group, cats had to have gingivitis, stomatitis, caudal mucositis, oral lesions and no systemic illness (Winer et al. 2016; Ploypetch et al. 2025). All cats in the study were evaluated for FeLV, FIV, FCV and FHV‐1 using antigen ELISA test. Blood samples were taken from cats with gingivostomatitis and serum was extracted. FeLV Ag/FIV Ab Combo antigen test (Bioguard, New Talpei City Taiwan) was applied to serum samples for the diagnosis of FeLV and FIV infection. For the diagnosis of FCV and FHV1 infection, Asan Easy Test FCV Ag and FHV antigen test (ASAN PHARMACEUTICAL CO. Lt. Korea) was performed.

### 2.3 Selection of Healthy Cats

Cats of different ages, breeds and sexes that were brought to the clinic for vaccination or general health checks, with normal routine clinical and laboratory (haemogram) examination results and negative antigen tests for the above diseases were considered healthy and included in the study.

### 2.4 Collection of Blood Samples

Blood samples with and without anticoagulant were taken once from all cats. Blood samples were taken from the vena cefana or cephalica. Blood samples with and without anticoagulant were collected once from all cats. Haemogram measurement was performed immediately. Blood samples collected for biomarker analysis were kept at room temperature for 15 min, centrifuged at 5000 rpm for 5 min, serum removed, and sera stored at −20°C until analysis. The biomarkers ET‐1, SDC‐1, MPO‐ANCA and IL‐6 were measured from the collected serum samples.

### 2.5 Treatment Protocol

Cats with gingivostomatitis received symptomatic treatment for gingivostomatitis and standard treatment for viral diseases. Oral antiseptics (Oral Cure, Yeniçağ Veterinary ECZA) and antibiotics were used to treat gingivostomatitis. Clindamycin (Klindan Bilim Pharmaceuticals) was administered at a dose of 2.2 mg/kg once daily for 10 days peros. Alpha‐interferon 30 U/day PO (ReferOn, Roche pharmaceuticals) was used as immunomodulator for 2 weeks and zidovudine (Retrovir, GlaxoSmithKline) 5 mg/kg for 10 days was used to prevent virus replication in cats with FeLV and FIV (Hartman 2017a). B complex, vitamin A, D, E and C were administered as supportive treatment.

### 2.6 Haemogram Analysis

WBC, RBC, Hb, Hct, MCV, MCHC and PLT parameters of venous blood samples collected from all cats were measured using the MS4e device (CFE 279, Haematology Analyser, Melet Schloesing Laboratories, France).

### 2.7 Biomarker Analysis

Serum concentrations of ET‐1, SDC‐1, MPO‐ANCA and IL‐6 in healthy cats and cats with gingivostomatitis were measured with cat‐specific ELISA test kits (Bioassay Technology Laboratory, Shanghai, China) using an absorbance microplate reader (ELx800 Absorbance Microplate Reader, USA) according to the manufacturer's instructions. For ET‐1 (Cat. No: E0095), the reported intra‐assay and inter‐assay CV were <8% and <10%, respectively, MDC was 1.18 ng/L, detection range 2–600 ng/L, SDC‐1 (Cat. No: E0068) reported intra‐assay (within‐study) and inter‐assay (between‐studies) CV <8% and <10%, respectively, MDC 0.025 ng/mL, detection range 0.05–30 ng/mL, MPO‐ANCA (Cat. No: E0070) reported intra‐assay and inter‐assay CV were <8% and <10%, MDC was 0.32 ng/mL, detection range was 0.5–150 ng/mL, intra‐assay and inter‐assay CV were <8% and <10%, MDC was 1.36 ng/L, detection range was 2–600 ng/L, respectively, for IL‐6 (Cat. No: E0029).

### 2.8 Statistical Analysis

In the evaluation of the data in this study, SPSS 25 statistical programme was used. Shapiro–Wilks test was applied to evaluate the prerequisites for normal distribution (parametric or nonparametric) of the data. As the study data showed a non‐parametric distribution, they were presented as medians (min/max). Mann–Whitney *U* test and Kruskal–Wallis test were used for comparison between groups. The significance level of the tests was accepted as *p* < 0.05.

## 3 Results

### 3.1 Clinical Finding

Cats with gingivostomatitis had anorexia, bad breath, gingivostomatitis, gingival hyperplasia, ulcers on the tongue and inside the mouth and mandibular lymphadenopathy. Medical treatment was applied to the sick cats. Although most cats responded well to treatment, recurrence was seen in some cases. Recurrence was seen in 15 cats with viral gingivostomatitis and 5 cats with gingivostomatitis of unknown cause. Recurrences occurred between 3 and 8 months. Of 55 cats with gingivostomatitis, 34 were viral infected and 21 were not viral infected. A total of 34 (61.82%) had a viral infection and 21 (38.18%) did not viral infection. The viral infections were 18 (52.94%) FeLV, 10 (29.41%) FIV, 5 (14.70%) FCV and 1 (2.94%) FHV (Table 1). Six of 55 cats with gingivostomatitis died.

### 3.2 Biomarker Finding

Biomarker concentrations of healthy cats and cats with gingivostomatitis are presented in Table 2, biomarker concentrations of healthy cats and cats with viral gingivostomatitis in Table 3, biomarker concentrations of cats with viral gingivostomatitis and cats with non‐viral gingivostomatitis in Table 4 and biomarker concentrations of cats with FeLV, FIV and FCV in Table 5. Cats with gingivostomatitis showed a statistically significant increase in ET‐1 (*p* ˂ 0.01) and SDC‐1 (*p* ˂ 0.001) concentrations compared to healthy cats, whereas no difference was observed in IL‐6 and MPO‐ANCA concentrations (*p* > 0.05) (Table 2). A statistically significant increase in ET‐1 (*p* ˂ 0.001) and SDC‐1 (*p* ˂ 0.01) concentrations, but no difference in IL‐6 and MPO‐ANCA (*p* > 0.05) concentrations were observed in cats with viral gingivostomatitis compared to healthy cats (Table 3). Cats with viral gingivostomatitis showed a significant increase in ET‐1 concentration (*p* ˂ 0.001) and no difference in SDC‐1, IL‐6 and MPO‐ANCA concentrations compared to cats with non‐viral gingivostomatitis (Table 4). No statistical difference was observed between the ET‐1, SDC‐1, MPO‐ANCA and IL‐6 parameters of cats with FeLV, FIV and FCV (*p* > 0.05) (Table 5).

**TABLE 2:** Biomarker levels of healthy cats and cats with gingivostomatitis (median, min–max).

Parameters	Healthy cats (n: 15)	Cats with gingivostomatitis (n: 55)	*p* value
ET‐1 (ng/L)	42.45 (14.37–56.65)	59.59 (22.67–157.34)	0.001
SDC‐1 (ng/mL)	3.76 (1.29–5.57)	4.81 (2.89–12.57)	0.000
MPO‐ANCA (ng/mL)	4.67 (3.27–9.65)	5.29 (3.09–13.65)	0.909
İL‐6 (ng/L)	112.78 (32.97–145.35)	113.14 (46.68–299.77)	0.601

*Note*: Significance level was accepted as *p* < 0.05.

Abbreviations: ET‐1, endothelin‐1; IL‐6, interleukin‐6; MPO‐ANCA, myeloperoxidase‐ANCA; SDC‐1, syndecan‐1.

**TABLE 3:** Biomarker levels of healthy cats and cats with viral gingivostomatitis (median, min–max).

Parameters	Healthy cats (n: 15)	Cats with viral gingivostomatitis (n: 34)	*p* value
ET‐1 (ng/L)	42.45 (14.37–56.65)	68.24 (34.78–157.34)	0.000
SDC‐1 (ng/mL)	3.76 (1.29–5.57)	4.76 (2.89–9.87)	0.001
MPO‐ANCA (ng/mL)	4.67 (3.27–9.65)	5.97 (3.09–13.65)	0.656
İL‐6 (ng/L)	112.78 (32.97–145.35)	117.49 (52.37–296.45)	0.340

*Note*: Significance level was accepted as *p* < 0.05.

Abbreviations: ET‐1, endothelin‐1; IL‐6, interleukin‐6; MPO‐ANCA, myeloperoxidase‐ANCA; SDC‐1, syndecan‐1.

**TABLE 4:** Biomarker levels of cats with viral and non‐viral gingivostomatitis (median, min–max).

Parameters	Cats with viral gingivostomatitis (n: 34)	Cats with non‐viral gingivostomatitis (n: 21)	*p* value
ET‐1 (ng/L)	68.24 (34.78–157.34)	46.65 (22.67–77.78)	0.001
SDC‐1 (ng/mL)	4.76 (2.89–9.87)	4.99 (3.47–12.57)	0.505
MPO‐ANCA (ng/mL)	5.97 (3.09–13.65)	4.67 (3.36–7.54)	0.174
İL‐6 (ng/L)	117.49 (52.37–269.45)	109.56 (46.68–299.77)	0.267

*Note*: Significance level was accepted as *p* < 0.05.

Abbreviations: ET‐1, endothelin‐1; IL‐6, interleukin‐6; MPO‐ANCA, myeloperoxidase‐ANCA; SDC‐1, syndecan‐1.

**TABLE 5:** Biomarker levels of FeLV, FIV and FCV in cats (median, min–max).

Parameter	Cats with FeLV (n: 17)	Cats with FIV (n: 10)	Cats with FCV (n: 6)
ET‐1 (ng/L)	68.99 (34.7–110.4)	60.11 (38.6–130.6)	99.02 (37.6–157.3)
SDC‐1 ng/mL)	5.01 (2.89–9.87)	4.66 (3.75–6.45)	5.24 (4.07–7.76)
MPO‐ANCA (ng/mL)	7.05 (3.11–13.65)	5.65 (3.09–9.45)	4.05 (3.09–6.66)
İL‐6 (ng/L)	113.06 (52.3–269.4)	110.60 (67.7–164.1)	121.54 (69.7–245.9)

*Note*: Significance level was accepted as *p* < 0.05.

Abbreviations: ET‐1, endothelin‐1; IL‐6, interleukin‐6; MPO‐ANCA, myeloperoxidase‐ANCA; SDC‐1, syndecan‐1.

### 3.3 Haemogram Finding

The haemogram parameters of healthy and gingivostomatitis cats are presented in Table 6, healthy and viral gingivostomatitis cats are presented in Table 7, viral and non‐viral gingivostomatitis cats are presented in Table 8 and FeLV, FIV and FCV cats are presented in Table 9. Although a statistically significant increase in WBC (*p* ˂ 0.05), GRA (*p* ˂ 0.05) and MCHC (*p* ˂ 0.01) was observed in cats with gingivostomatitis compared to healthy cats, a decrease in MCV (*p* ˂ 0.05) and Hct (*p* ˂ 0.01) levels, and no difference was observed for LYM, RBC, Hb and PLT parameters (*p* > 0.05) (Table 6). Statistically significant increase in WBC (*p* ˂ 0.01), MON (*p* ˂ 0.01), GRA (*p* ˂ 0.05) and MCHC (*p* ˂ 0.01) levels in cats with viral gingivostomatitis compared to healthy cats, decrease in MCV (*p* ˂ 0.05) and Hct (*p* ˂ 0.05) levels, and no difference was observed for LYM, RBC, Hb and PLT parameters (*p* > 0.05) (Table 7). Statistically significant increase in WBC (*p* ˂ 0.01) and MON (*p* ˂ 0.01) in cats with viral gingivostomatitis compared to cats with non‐viral gingivostomatitis, decrease in MCV (*p* ˂ 0.05) and Hct (*p* ˂ 0.05), and no difference was observed for GRA, LYM, RBC, MCV, MCHC, Hb and PLT parameters (*p* ˂ 0.05) (Table 8). No statistical difference was observed between WBC, MON, GRA, LYM, RBC, MCV, MCHC, Hb and PLT parameters of cats with FeLV, FIV and FCV (*p* > 0.05) (Table 9).

**TABLE 6:** Means and significance (median, min–max) of haemogram parameters of healthy cats and cats with gingivostomatitis.

Parameters	Healthy cats (n: 15)	Cats with gingivostomatitis (n: 55)	*p* value
WBC (m/mm^3^)	9.13 (4.37–16.06)	12.34 (3.70–47.91)	0.016
LYM (m/mm^3^)	4.12 (1.19–12.47)	3.98 (0.55–9.83)	0.582
MON (m/mm^3^)	0.35 (0.01–1.12)	0.41 (0.05–3.32)	0.190
GRA (m/mm^3^)	4.23 (2.24–7.33)	6.97 (1.38–42.33)	0.016
RBC (m/mm^3^)	10.81 (7.54–12.77)	10.15 (3.26–13.94)	0.193
MCV (fL)	51.00 (38.00–59.00)	42.00 (27.00–74.00)	0.010
Hct (%)	54.20 (37.60–64.90)	41.70 (22.60–84.80)	0.001
MCHC (g/dL)	27.50 (22.20–35.60)	33.60 (19.40–38.00)	0.002
Hb (g/dL)	14.50 (11.00–16.80)	14.10 (5.50–26.30)	0.492
PLT (m/mm^3^)	390.00 (68.00–489.00)	399.00 (38.00–798.00)	0.621

*Note*: Significance level was accepted as *p* < 0.05.

Abbreviations: GRA, granulocytes; Hb, haemoglobin; Hct, haematocrit; LYM, lymphocytes; MCHC, mean erythrocyte haemoglobin concentration; MCV, mean erythrocyte volume; MON, monocytes; PLT, platelets; RBC, erythrocytes; WBC, total leukocytes.

**TABLE 7:** Means and significance (median, min–max) of haemogram parameters of healthy cats and cats with viral gingivostomatitis.

Parameters	Healthy cats (n: 15)	Cats with viral gingivostomatitis (n: 34)	*p* value
WBC (m/mm^3^)	9.13 (4.37–16.06)	13.46 (3.70–47.91)	0.001
LYM (m/mm^3^)	4.12 (1.19–12.47)	4.28 (0.58–9.83)	0.795
MON (m/mm^3^)	0.35 (0.01–1.12)	0.60 (0.05–3.32)	0.048
GRA (m/mm^3^)	4.23 (2.24–7.33)	7.66 (1.38–42.33)	0.002
RBC (m/mm^3^)	10.81 (7.54–12.77)	10.09 (4.66–13.94)	0.220
MCV (fL)	51.00 (38.00–59.00)	42.50 (27.00–74.00)	0.036
Hct (%)	54.20 (37.60–64.90)	41.75 (22.60–84.80)	0.005
MCHC (g/dL)	27.50 (22.20–35.60)	38.95 (19.40–38.00)	0.004
Hb (g/dL)	14.50 (11.00–16.80)	14.05 (7.90–26.30)	0.416
PLT (m/mm^3^)	390.00 (68.00–489.00)	393.00 (38.00–798.00)	0.494

*Note*: Significance level was accepted as *p* < 0.05.

Abbreviations: GRA, granulocytes; Hb, haemoglobin; Hct, haematocrit; LYM, lymphocytes; MCHC, mean erythrocyte haemoglobin concentration; MCV, mean erythrocyte volume; MON, monocytes; PLT, platelets; RBC, erythrocytes; WBC, total leukocytes.

**TABLE 8:** Mean and significance (median, min–max) of haemogram parameters of cats with viral and non‐viral gingivostomatitis.

Parameter	Cats with viral gingivostomatitis (n: 34)	Cats with non‐viral gingivostomatitis (n: 21)	*p* value
WBC (m/mm^3^)	13.46 (3.70–47.91)	9.95 (4.97–21.88)	0.004
LYM (m/mm^3^)	4.28 (0.58–9.83)	3.38 (0.55–9.00)	0.703
MON (m/mm^3^)	0.60 (0.05–3.32)	0.33 (0.06–0.69)	0.003
GRA (m/mm^3^)	7.66 (1.38–42.33)	5.30 (1.81–17.16)	0.023
RBC (m/mm^3^)	10.09 (4.66–13.94)	10.20 (3.26–12.36)	0.579
MCV (fL)	42.50 (27.00–74.00)	40.00 (36.00–69.00)	0.755
Hct (%)	41.75 (22.60–84.80)	41.70 (22.60–60.00)	0.522
MCHC (g/dL)	33.95 (19.40–38.00)	33.40 (24.50–37.60)	0.628
Hb (g/dL)	14.05 (7.90–26.30)	14.40 (5.50–17.30)	0.697
PLT (m/mm^3^)	393.50 (38.00–798.00)	399.00 (146.00–627.00)	0.591

*Note*: Significance level was accepted as *p* < 0.05.

Abbreviations: GRA, granulocytes; Hb, haemoglobin; Hct, haematocrit; LYM, lymphocytes; MCHC, mean erythrocyte haemoglobin concentration; MCV, mean erythrocyte volume; MON, monocytes; PLT, platelets; RBC, erythrocytes; WBC, total leukocytes.

**TABLE 9:** Means and significance of haemogram parameters of FeLV, FIV and FCV cats (median, min–max).

Parameters	Cats with FeLV (n: 17)	Cats with FIV (n: 10)	Cats with FCV (n: 6)
WBC (m/mm^3^)	15.34 (6.61–47.91)	12.49 (3.70–24.57)	11.63 (6.73–19.70)
LYM (m/mm^3^)	4.57 (0.80–8.50)	2.52 (0.58–9.83)	5.55 (2.75–7.32)
MON (m/mm^3^)	0.68 (0.07–3.32)	0.55 (0.08–1.21)	0.73 (0.05–0.90)
GRA (m/mm^3^)	8.93 (2.73–42.33)	6.76 (1.38–21.64)	4.65 (3.50–13.25)
RBC (m/mm^3^)	10.26 (4.66–13.54)	9.80 (5.52–11.26)	11.40 (9.72–13.94)
MCV (fL)	41.50 (34.00–65.00)	44.50 (27.00–61.00)	55.00 (32.00–74.00)
Hct (%)	42.30 (23.10–82.60)	39.10 (22.60–63.30)	66.20 (37.50–84.80)
MCHC (g/dL)	33.45 (21.50–38.00)	34.05 (23.00–36.80)	34.10 (19.40–37.20)
Hb (g/dL)	13.60 (7.90–18.50)	13.80 (8.20–18.40)	16.40 (13.60–26.30)
PLT (m/mm^3^)	307.00 (84.00–747.00)	458.00 (38.00–798.00)	451.00 (108.00–526.00)

*Note*: Significance level was accepted as *p* < 0.05.

Abbreviations: GRA, granulocytes; Hb, haemoglobin; Hct, haematocrit; LYM, lymphocytes; MCHC, mean erythrocyte haemoglobin concentration; MCV, mean erythrocyte volume; MON, monocytes; PLT, platelets; RBC, erythrocytes; WBC, total leukocytes.

## 4 Discussion

Feline gingivostomatitis is an immune‐mediated inflammatory disease of the oral mucosa caused by the excessive release and imbalance of inflammatory cytokines. The most common clinical sign is chronic gingivostomatitis (Hurley and Sykes 2003; Kuek et al. 2007; Lee et al. 2020; Ploypetch et al. 2025). Viral diseases, bacterial plaque, gum disease, environmental factors and breeds play an important role in chronic gingivostomatitis of cats (Kuek et al. 2007; Kim et al. 2023; Soltero‐Rivera et al. 2023). Viral infections such as FeLV, FIV, FCV and FHV‐1 have been reported to be particularly effective in the development of gingivostomatitis (Lutz et al. 2009; Winer et al. 2016; Nelson and Couto 2019). Oral mucosal biopsy samples from 27 cats with chronic gingivostomatitis were examined and FeLV antigen was detected immunohistochemically in the mucosal epithelium in 30% of the cats (Lutz et al. 2009). This study found ptyalism, anorexia, halitosis, gingivitis and stomatitis, and in some cases gingival hyperplasia, tongue ulceration and mandibular lymphadenopathy in cats with gingivostomatitis. Cats with gingivostomatitis were treated and the lesions healed. However, relapses were observed in 15 cats with viral gingivostomatitis and 5 cats with non‐viral gingivostomatitis. Relapse occurred between 3 and 8 months. Of 55 cats with gingivostomatitis, 34 (61.82%) had viral infections and 21 (38.18%) had non‐viral infections. The viral infections causing gingivostomatitis in cats were FeLV in 18 (52.94%), FIV in 10 (29.41%), FCV in 5 (14.70%) and FHV in 1 (2.94%) (Table 1). Our clinical findings were consistent with the clinical findings of many researchers (Hurley and Sykes 2003; Druet and Hennet 2017; Kim et al. 2023; Soltero‐Rivera et al. 2023). As reported by many researchers (Lee et al. 2010; Quimby et al. 2008; Lee et al. 2020), viral infections such as FeLV, FIV, FCV and FHV are effective in the aetiology of feline gingivostomatitis. In the present study, the aetiology of 21 cats with gingivostomatitis could not be determined. We think that multifactors such as different bacteria, dental plaque, hypersensitivity, gingival diseases, environmental stressors and breeds are effective in the aetiology of gingivostomatitis in these cats.

EGC damage may occur in the vessels as a result of inflammation in the mouth and gums in cats with gingivostomatitis caused by viral infections and other factors. This study selected SDC‐1, MPO‐ANCA, ET‐1 and IL‐6 as biomarkers because SDC‐1 and ET‐1 can accurately determine EGC damage, MPO‐ANCA can detect immune‐mediated vasculitis and IL‐6 can determine inflammation. In this study, the first time that EGC damage markers have been used to demonstrate endothelial damage in the structure surrounding the lumen surface of the oral vascular system in cats with gingivostomatitis of various origins. ET‐1 and SDC‐1 markers were found to be important for diagnosing EGC damage in gingivostomatitis.

ET‐1 has been found to increase ET‐1 levels in pneumonia, pulmonary hypertension, interstitial fibrosis and acute respiratory distress syndrome in humans (Bouallegue et al. 2007). An increase in serum ET‐1 concentration was observed in premature calves with respiratory distress compared to premature calves without respiratory distress and healthy calves, and the ET‐1 biomarker was found to be prognostic for mortality in premature calves (Ider et al. 2021). İsmailoğlu (2023) found a significant increase in ET‐1 concentration in acute respiratory distress syndrome in cats and associated this increase with damage to the lung epithelium and endothelium. Ider et al. (2024) reported that SDC‐1 and ET‐1 can be used as diagnostic and serum ET‐1 and VEGF‐A can be used as prognostic biomarkers in cats with haemotropic mycoplasmosis. In the present study, the serum ET‐1 concentration in cats with gingivostomatitis was higher in cats with viral gingivostomatitis (Table 2) than in healthy cats (*p* ˂ 0.01) compared to healthy cats (Table 3) and a statistically significant increase in serum ET‐1 concentration was observed in cats with viral gingivostomatitis (*p* ˂ 0.001) compared to cats with non‐viral gingivostomatitis (Table 4). In addition, no statistically significant difference in serum ET‐1 concentration was observed between cats with FeLV, FIV and FCV (*p* > 0.05) (Table 5). The results of this study were in agreement with the results of many researchers (Bouallegue et al. 2007; Ider et al. 2021; Ismailoglu 2023; Ider et al. 2024). The increase in serum ET‐1 concentration in cats with gingivostomatitis was evaluated as an indicator of the development of EGC damage in the oral vasculature associated with oral inflammation from other causes, including viral infections. We believe that the ET‐1 marker may have a reliable diagnostic value in detecting EGC damage in patients with gingivostomatitis.

Increased serum SDC‐1 concentration has been shown to be the main indicator of EGC damage in humans and rats (Torres‐Filho et al. 2016; Barber et al. 2021; Reszagi et al. 2022). Naseri et al. (2020) reported that no difference was observed in SDK‐1 concentration in dogs with parvoviral enteritis compared to healthy dogs. Ider et al. (2024) reported that serum levels of SDC‐1 and ET‐1 were significantly increased in cats with haemotropic mycoplasmosis and that this marker can be used as a diagnostic and serum ET‐1 and VEGF‐A as prognostic biomarkers. In the present study, there was a statistically significant increase in serum SDC‐1 concentration (*p* ˂ 0.001) in cats with gingivostomatitis compared to healthy cats (Table 2) and in serum SDC‐1 concentration (*p* ˂ 0.01) in cats with viral gingivostomatitis compared to healthy cats (Table 3). However, no statistical difference was observed in serum SDC‐1 concentration of cats with viral gingivostomatitis compared to cats with non‐viral gingivostomatitis (*p* > 0.05) (Table 4) and between cats with FeLV, FIV and FCV (*p* > 0.05) (Table 4). The results of this study were in agreement with the results reported by many researchers (Rehm et al. 2007; Torres‐Filho et al. 2016; Barber et al. 2021; Ider et al. 2024). The increase in serum SDC‐1 concentration in cats with gingivostomatitis was evaluated as an indicator of oral EGC damage related to inflammation in the oral region due to viral infections and other causes. Increased serum SDC‐1 concentration have been associated with EGC damage as well as endothelial matrix remodelling and repair of EGC damage. Like many others (Cao et al. 2019; Naseri et al. 2020; Barber et al. 2021), we believe that SDC‐1 and ET‐1 are highly reliable biomarkers of diagnostic importance in determining EGC damage.

PR3‐ANCA and MPO‐ANCA concentrations are reported to increase in vasculitis (Schönermarck et al. 2015). Although PR3‐ANCA level increases in granulomatous inflammation of the respiratory system and organs other than the kidney, MPO‐ANCA level increases mostly in kidney diseases (Quintana et al. 2014). Gulersoy et al. (2023) determined that no difference was observed in serum MPO‐ANCA and PR3‐ANCA concentrations in cats with FIP compared to healthy cats. In the present study, serum MPO‐ANCA (*p* > 0.05) concentrations of cats with gingivostomatitis (Table 2) and cats with viral gingivostomatitis (Table 3) were higher than those of healthy cats (*p* > 0.05), and serum MPO‐ANCA (*p* > 0.05) concentration in cats with viral gingivostomatitis compared to cats with non‐viral gingivostomatitis (Table 4), and no statistical difference in serum MPO‐ANCA (*p* > 0.05) concentration was observed between cats with FeLV, FIV and FCV (Table 5). The results of this study were not consistent with those of Quintana et al. (2014) but were consistent with those of Gulersoy et al. 2023). The possible reason why our results do not agree with those of Quintana et al. (2014) is related to the fact that granulomatous vasculitis does not occur in gingivostomatitis, as well as the absence of extensive vasculitis. Although vasculitis occurs in FIP cats, serum MPO‐ANCA levels do not increase (Gulersoy et al. 2023), and the reason for this is the absence of extensive vasculitis in the organism, which supports our view.

It has been reported that IL‐6 levels increase in sepsis and acute organ injury and that IL‐6 can be used as a prognostic biomarker (Vaporidi et al. 2008). It has also been suggested that IL‐6 can be used to determine prolonged mechanical ventilation, organ dysfunction, morbidity and mortality in patients with acute respiratory distress syndrome (Darif et al. 2021). Increased IL‐6 level in patients with sepsis is accepted as an indicator of the development of acute lung and organ damage (O'Malley and Moldawer 2006). In the present study, serum IL‐6 concentrations of cats with gingivostomatitis (Table 2) and cats with viral gingivostomatitis (*p* ˂ 0.05) were higher than those of healthy cats (Table 3), and the serum IL‐6 concentrations of cats with viral gingivostomatitis (*p* > 0.05) were higher than those of cats with non‐viral gingivostomatitis (Table 4), and no statistical difference in serum IL‐6 concentrations (*p* > 0.05) was observed between cats with FeLV, FIV and FCV (Table 5). In acute inflammation, IL‐6 levels increase, activating the immune system and initiating a strong inflammatory process, and in later stages, IL‐6 levels decrease and return to normal levels (Vaporidi et al. 2008; Wiegertjes et al. 2019; Darif et al. 2021). The possible reason for the lack of increase in serum IL‐6 concentration in cats with gingivostomatitis can be explained by the chronic nature of the inflammation. IL‐6 is a pro‐inflammatory marker that is released in peracute and acute periods (Darif et al. 2021).

The leukogram change in viral disease is a decrease in the number of lymphocytes (Jiang et al. 2020). Anaemia and lymphopenia have been reported to be the most common haematological findings in FeLV‐positive cats (Beatty et al. 2011; da Costa et al. 2017; Biezus et al. 2019). It was reported that erythropenia was observed in 50% of FeLV‐positive cats and 40% of FIV‐positive cats, and leucocytosis was observed in 25% of FeLV‐positive cats and 40% of FIV‐positive cats (Rudan et al. 2017). Shelton et al. (1990) found anaemia (36%), lymphopenia (53%), neutropenia (34%) and thrombocytopenia (8%) in FIV‐positive cats. Erythropenia was observed in 32% of FeLV‐positive cats and 23% of FIV‐positive cats, and leucocytosis in 36% of FeLV‐positive cats and 20% of FIV‐positive cats (Biezus et al. 2023). Although haematological abnormalities such as anaemia, thrombocytopenia, neutropenia and lymphocytosis were observed in FeLV‐positive cats, no haematological abnormalities were reported in FIV‐positive cats (Gleich and Hartmann 2009). Anaemia and lymphopenia were observed as haematological abnormalities in FeLV‐ and FIV‐positive young cats, and these abnormalities were more common in FeLV‐positive cats than in FIV‐positive cats (Biezus et al. 2023). In the present study, there was a statistically significant increase in WBC (*p* ˂ 0.05) and GRA (*p* ˂ 0.05) counts of cats with gingivostomatitis compared to healthy cats (Table 6), and a statistically significant increase in WBC (*p* ˂ 0.01), MON (*p* ˂ 0.01) and GRA (*p* ˂ 0.05) counts of cats with viral gingivostomatitis compared to healthy cats (Table 7). In addition, there was a statistically significant increase in WBC (*p* ˂ 0.01) and MON (*p* ˂ 0.01) counts in cats with viral gingivostomatitis compared to cats with non‐viral gingivostomatitis (Table 8), whereas no difference was observed in LYM (*p* > 0.05). In addition, no statistically significant difference was found between WBC, MON, GRA and LYM counts (*p* > 0.05) in cats with FeLV, FIV and FCV (Table 9). Our results were not in agreement with the results of many researchers (Shelton et al. 1990; Beatty et al. 2011; da Costa et al. 2017; Biezus et al. 2019) but were in agreement with the results of many researchers (Rudan et al. 2017; Biezus et al. 2023). The reason for the significant increase in WBC and GRA counts in cats with gingivostomatitis compared to healthy cats is related to the fact that the cellular response to antigens is always given through GRA (band neutrophil increase) (Hartmann 2017b; Nelson and Couto 2019). The increase in MON, especially in cats with viral gingivostomatitis, can be explained by the cellular response to viruses via MON (Nelson and Couto 2019). We believe that the possible reason why lymphopenia does not develop in cats with viral gingivostomatitis is related to the absence of an acute viral infection phase. This is because FeLV and FIV infections in cats are chronic and leukopenia does not occur unless immunosuppression develops.

However, the study has several limitations: (1) cats with gingivostomatitis were not histopathologically examined for glycocalyx damage, (2) the cat population included in the study was small and (3) a limited number of glycocalyx biomarker measurements were performed due to lack of financial budget. All these issues need to be addressed in further studies.

In conclusion, FeLV, FIV and FCV infections played an important role in the aetiology of feline gingivostomatitis, but other causes were also effective. In cats with gingivostomatitis, serum concentrations of ET‐1 and SDC‐1 were increased, whereas no difference was found in MPO‐ANCA and IL‐6 concentrations. It has been shown that EGC damage occurs in cats with gingivostomatitis, and ET‐1 and SDC‐1 biomarkers have reliable diagnostic value in the detection of this damage.

## Author Contributions

**Saadet Gözde Korkmaz**: conceptualization, methodology, investigation, writing – original draft, writing – review and editing, data curation. **Mahmut Ok**: conceptualization, methodology, data curation, investigation, writing – original draft, writing – review and editing, project administration. All authors contributed to the article and approved the submitted version.

## Ethics Statement

The study was conducted with the approval of the Selcuk University Veterinary Faculty Experimental Animal Production and Research Center Ethics Committee with decision number 2024/008.

## Conflicts of Interest

The authors declare no conflicts of interest.

## Peer Review

The peer review history for this article is available at https://www.webofscience.com/api/gateway/wos/peer‐review/10.1002/vms3.70525.

## Data Availability

All study data are presented in the article.
